# Supplementary material for: Head-to-head comparison of [¹⁸F]F-PSMA-1007 PET/CT and [⁶⁸Ga]Ga-PSMA-11 PET/CT for intraprostatic tumour detection and localisation using histopathology as reference: A prospective single-centre diagnostic accuracy study
Source: Eur J Nucl Med Mol Imaging. 2026 Sep 19;53(13):8640–50. doi: 10.1007/s00259-026-08191-9 (PMC13727829; doi:10.1007/s00259-026-08191-9)
Supplement: Supplementary file 1 [file 259_2026_8191_MOESM1_ESM.docx]

**Supplementary material**

**Figure S1.** A prostate pathology segmentation worksheet (A) was used to register tumour foci on each histological slice to the corresponding anatomical region. To ensure standardised reporting, a six-segment model of the prostate gland (B) was applied. All findings were mapped to six regions: right base (RB), left base (LB), right mid-gland (RM), left mid-gland (LM), right apex (RA), and left apex (LA). The presence of carcinoma on any single slice rendered the corresponding region positive, with localisation recorded as right or left.

**Figure S2.** Schematic illustrating the neighbouring approach used to translate prostate imaging segmentation worksheets into sextant regions across all three modalities. Example 1 shows three tumour foci—left base (blue), right mid-gland (black), and apical (orange)—translated to left base, right mid-gland, and bilateral apex positivity. Example 2 shows foci in the cranial right mid-gland (green) and caudal left mid-gland (red), corresponding to right mid-gland/right base and left mid-gland/left apex involvement, respectively.

| Left apex |  |  |  |  |  |  |
| --- | --- | --- | --- | --- | --- | --- |
| Prevalence = 0.7200 | Sensitivity (95% CI) | Specificity (95% CI) | AUC (95% CI) | Accuracy (95% CI) | PPV (95% CI) | NPV (95% CI) |
| F-PSMA PET/CT | 66.7 (51.2, 82.1) | 71.4 (47.7, 95.2) | 69.0 (54.9, 83.2) | 68.0 (55.0, 81.0) | 85.7 (75.1, 96.3) | 45.5 (31.3, 59.6) |
| Ga-PSMA PET/CT | 61.1 (45.1, 77.1) | 71.4 (47.7, 95.2) | 66.3 (52.0, 80.6) | 64.0 (50.7, 77.3) | 84.6 (73.3, 96.0) | 41.7 (28.8, 54.5) |
| mpMRI | 50.0 (32.6, 67.4) | 69.2 (44.1, 94.4) | 59.6 (44.3, 74.9) | 55.4 (41.0, 69.7) | 80.7 (66.8, 94.5) | 35.0 (23.6, 46.4) |
| Left basal |  |  |  |  |  |  |
| Prevalence = 0.5400 | Sensitivity (95% CI) | Specificity (95% CI) | AUC (95% CI) | Accuracy (95% CI) | PPV (95% CI) | NPV (95% CI) |
| F-PSMA PET/CT | 59.3 (40.7, 77.9) | 69.6 (50.7, 88.4) | 64.4 (51.2, 77.7) | 64.0 (50.7, 77.3) | 69.6 (54.9, 84.3) | 59.3 (46.4, 72.1) |
| Ga-PSMA PET/CT | 44.4 (25.6, 63.3) | 60.9 (40.9, 80.9) | 52.7 (38.9, 66.4) | 52.0 (38.3, 65.7) | 57.1 (40.9, 73.4) | 48.3 (36.5, 60.1) |
| mpMRI | 8.3 (-2.8, 19.4) | 90.5 (77.9, 103.1) | 49.4 (41.0, 57.8) | 46.1 (37.8, 54.5) | 50.7 (3.8, 97.6) | 45.7 (41.1, 50.3) |
| Left mid |  |  |  |  |  |  |
| Prevalence = 0.8400 | Sensitivity (95% CI) | Specificity (95% CI) | AUC (95% CI) | Accuracy (95% CI) | PPV (95% CI) | NPV (95% CI) |
| F-PSMA PET/CT | 76.2 (63.3, 89.1) | 50.0 (15.2, 84.8) | 63.1 (44.5, 81.6) | 72.0 (59.8, 84.2) | 88.9 (81.8, 96.0) | 28.6 (10.6, 46.6) |
| Ga-PSMA PET/CT | 85.7 (75.1, 96.3) | 50.0 (15.2, 84.8) | 67.9 (49.7, 86.0) | 80.0 (69.5, 90.5) | 90.0 (83.6, 96.4) | 40.0 (15.6, 64.4) |
| mpMRI | 63.2 (47.8, 78.5) | 71.4 (37.8, 105.0) | 67.3 (48.8, 85.8) | 64.5 (50.5, 78.5) | 92.1 (83.3, 100.8) | 27.0 (14.6, 39.4) |
| Right apex |  |  |  |  |  |  |
| Prevalence = 0.7000 | Sensitivity (95% CI) | Specificity (95% CI) | AUC (95% CI) | Accuracy (95% CI) | PPV (95% CI) | NPV (95% CI) |
| F-PSMA PET/CT | 65.7 (49.9, 81.5) | 66.7 (42.7, 90.6) | 66.2 (51.9, 80.5) | 66.0 (52.8, 79.2) | 82.1 (71.0, 93.3) | 45.5 (31.0, 59.9) |
| Ga-PSMA PET/CT | 45.7 (29.2, 62.3) | 73.3 (50.9, 95.8) | 59.5 (45.6, 73.5) | 54.0 (40.6, 67.4) | 80.0 (65.3, 94.7) | 36.7 (26.6, 46.7) |
| mpMRI | 45.2 (27.6, 62.7) | 85.7 (67.3, 104.1) | 65.4 (52.7, 78.2) | 57.3 (43.8, 70.8) | 88.1 (73.9, 102.2) | 40.1 (30.8, 49.4) |
| Right basal |  |  |  |  |  |  |
| Prevalence = 0.5000 | Sensitivity (95% CI) | Specificity (95% CI) | AUC (95% CI) | Accuracy (95% CI) | PPV (95% CI) | NPV (95% CI) |
| F-PSMA PET/CT | 60.0 (40.7, 79.3) | 68.0 (49.7, 86.3) | 64.0 (50.7, 77.3) | 64.0 (50.7, 77.3) | 65.2 (50.3, 80.1) | 63.0 (50.1, 75.8) |
| Ga-PSMA PET/CT | 64.0 (45.1, 82.9) | 80.0 (64.3, 95.7) | 72.0 (59.7, 84.3) | 72.0 (59.7, 84.3) | 76.2 (60.9, 91.4) | 69.0 (57.0, 81.0) |
| mpMRI | 38.1 (17.3, 58.9) | 95.8 (87.8, 103.9) | 67.0 (55.8, 78.1) | 67.0 (55.8, 78.1) | 90.1 (72.4, 107.9) | 60.8 (52.5, 69.0) |
| Right mid |  |  |  |  |  |  |
| Prevalence = 0.8000 | Sensitivity (95% CI) | Specificity (95% CI) | AUC (95% CI) | Accuracy (95% CI) | PPV (95% CI) | NPV (95% CI) |
| F-PSMA PET/CT | 82.5 (70.7, 94.3) | 50.0 (18.9, 81.1) | 66.3 (49.6, 82.9) | 76.0 (64.7, 87.3) | 86.8 (79.5, 94.1) | 41.7 (19.4, 64.0) |
| Ga-PSMA PET/CT | 85.0 (73.9, 96.1) | 40.0 (9.5, 70.5) | 62.5 (46.3, 78.7) | 76.0 (65.2, 86.8) | 85.0 (78.3, 91.7) | 40.0 (14.5, 65.5) |
| mpMRI | 68.6 (53.1, 84.0) | 90.0 (71.3, 108.7) | 79.3 (67.2, 91.4) | 72.9 (60.0, 85.8) | 96.5 (90.1, 102.9) | 41.7 (28.8, 54.7) |

**Table S1.** Diagnostic accuracy parameters for each index test were calculated per region, with whole-mount histopathology as the reference standard for intraprostatic tumour detection, treating each region as an independent unit.

CI = confidence interval; AUC = area under ROC curve; PPV = positive predictive value; NPV = negative predictive value.

**Table S2.** Estimates of diagnostic accuracy parameters for each index test for the detection of clinically significant prostate cancer were obtained from region-based analyses using mixed-effects regression models to account for repeated measurements within patients, with all ISUP grade group 2 lesions considered positive.

| Index test | Sensitivity  (95% CI) | Specificity  (95% CI) | AUC  (95% CI) | Accuracy (95% CI) | PPV  (95% CI) | NPV  (95% CI) |
| --- | --- | --- | --- | --- | --- | --- |
| [¹⁸F]F-PSMA-1007 N=300 | 67.9 (60.0, 75.9) | 71.8 (58.7, 84.8) | 69.8 (62.6, 77.1) | 68.8 (62.3, 75.4) | 88.7 (84.1, 93.4) | 40.7 (33.6, 47.7) |
| [⁶⁸Ga]Ga-PSMA-11 N=300 | 63.6 (56.2, 71.0) | 68.9 (55.3, 82.5) | 66.2 (59.2, 73.3) | 64.8 (59.0, 70.7) | 87.0 (82.1, 91.8) | 36.7 (30.9, 42.5) |
| mpMRI  N=270 | 47.8 (40.8, 54.8) | 93.6 (85.2, 101.9) | 70.7 (64.8, 76.6) | 58.5 (52.5, 64.6) | 96.0 (91.0, 101.1) | 35.4 (31.5, 39.4) |

N = total number; CI = confidence interval; AUC = area under ROC curve; PPV = positive predictive value; NPV = negative predictive value

**Table S3.** Pairwise comparison of sensitivity and specificity of [¹⁸F]F-PSMA PET/CT, [⁶⁸Ga]Ga-PSMA PET/CT, and mpMRI for detection of intraprostatic tumour using a region-based mixed-effects regression model accounting for repeated measurements within patients, with all ISUP grade group 2 lesions considered positive.

| Comparison (Differences) | Difference in sensitivity (95% CI), P | Difference in specificity (95% CI), P |
| --- | --- | --- |
| [¹⁸F]F-PSMA-1007 vs [⁶⁸Ga]Ga-PSMA-11 | 0.043 (-0.012, 0.099), 0.12 | 0.029 (-0.158, 0.215), 0.76 |
| [¹⁸F]F-PSMA-1007 PET/CT vs mpMRI | 0.201 (0.106, 0.297), < 0.001 | -0.218 (-0.362, -0.075), 0.003 |
| [⁶⁸Ga]Ga-PSMA-11 PET/CT vs mpMRI | 0.158 (0.073, 0.243), < 0.001 | -0.247 (-0.393, -0.101), < 0.001 |

CI = confidence interval; P = probability value
