# Supplementary material for: Head-to-head comparison of [¹⁸F]F-PSMA-1007 PET/CT and [⁶⁸Ga]Ga-PSMA-11 PET/CT for intraprostatic tumour detection and localisation using histopathology as reference: A prospective single-centre diagnostic accuracy study
Source: Eur J Nucl Med Mol Imaging. 2026 Sep 19;53(13):8640–50. doi: 10.1007/s00259-026-08191-9 (PMC13727829; doi:10.1007/s00259-026-08191-9)
Supplement: Supplementary file 3 [file 259_2026_8191_MOESM3_ESM.docx]

**Title Page**

**Title: Head-to-Head Comparison of [¹⁸F]F-PSMA-1007 PET/CT and [⁶⁸Ga]Ga-PSMA-11 PET/CT for Intraprostatic Tumor Detection** **and Localisation Using Histopathology as Reference: A Prospective Single-centre Diagnostic Accuracy Study**

**Running title:** Intraprostatic Tumor Detection by Imaging

Farid Gossili^1,2,3,*^, Frederik Harving^4^, Astrid Christine Petersen^4^, Claus Madsen^5^, Kirsten Bouchelouche^6^, Niels Henrik Bruun^7^, Robert Johannes Leusink^8^, Ayesha Ahmed^8^, Anja Morratz Laursen^9^, Helle D. Zacho^1,2^

^1^Department of Nuclear Medicine and Clinical Cancer Research Center, Aalborg University Hospital, Aalborg, Denmark

^2^ Department of Clinical Medicine, Aalborg University, Aalborg, Denmark

^3^Department of Clinical Physiology, Viborg Regional Hospital, Viborg, Denmark

^4^Department of Pathology, Aalborg University Hospital, Aalborg, Denmark

^5^Department of Nuclear Medicine, Copenhagen University Hospital Herlev and Gentofte, Herlev, Denmark

^6^Department of Nuclear Medicine, Aarhus University Hospital, Aarhus, Denmark

^7^Research Data and Biostatistics, Aalborg University Hospital, Aalborg, Denmark

^8^Department of Radiology, Aalborg University Hospital, Aalborg, Denmark

^9^Department of Urology, Aalborg University Hospital, Aalborg, Denmark

^*^Corresponding author: Email: [f.gossili@rn.dk](mailto:f.gossili@rn.dk), Tel/Fax: +45 97 66 55 00, Address: Aalborg University Hospital, Hospitalsbyen, Røntgen- og Scanningshuset, Entrance 6, Hospitalsbyen 1, 9260 Gistrup, Floor 0, Denmark, ORCID ID: 0000-0003-4029-3197

**Abstract**

**Purpose**

Prostate-specific membrane antigen (PSMA) PET has emerged as a sensitive imaging modality for prostate cancer (PC), but its role in intraprostatic tumour localisation relative to multiparametric MRI (mpMRI) and differences between [¹⁸F]- and [⁶⁸Ga]-PSMA ligands remain unclear. This study compared the diagnostic performance of [¹⁸F]F-PSMA-1007 PET/CT, [⁶⁸Ga]Ga-PSMA-11 PET/CT, and mpMRI for intraprostatic tumour localisation and detection of clinically significant prostate cancer (csPC).

**Methods**

In this prospective single-centre study, patients with biopsy-proven high-risk PC undergoing radical prostatectomy were recruited. All patients underwent [¹⁸F]F-PSMA-1007 PET/CT and [⁶⁸Ga]Ga-PSMA-11 PET/CT. Following a protocol amendment mpMRI was added as a secondary exploratory imaging modality and performed in a subset of patients prior to surgery. Histopathology was reference standard; csPC was defined as ≥ISUP3 or ISUP2 with tertiary Gleason 5 pattern. Diagnostic performance was assessed per-patient and per-region levels, dividing the prostate into sextants and using mixed regression models to account for clustering. Secondary analyses evaluated the detection of pathological features beyond the prostate, including extraprostatic extension (EPE) and seminal vesicle invasion (SVI).

**Results**

Fifty patients were included in the analysis of PSMA PET/CT, 45 patients had mpMRI. At the patient level, [¹⁸F]F-PSMA-1007 PET/CT, [⁶⁸Ga]Ga-PSMA-11 PET/CT and mpMRI demonstrated sensitivities of 98%, 100%, and 96%, respectively, for the detection of csPC, with corresponding diagnostic accuracies of 96%, 98%, and 93%, respectively. Of 300 sextants, 205 contained csPC. [¹⁸F]F-PSMA-1007 PET/CT identified csPC in 143/300 sextants (accuracy: 69.8%, 95%CI 64.0–75.6). [⁶⁸Ga]Ga-PSMA-11 PET/CT detected csPC in 136/300 sextants (accuracy: 67.8% 95%CI 62.3–73.4). mpMRI identified csPC in 89/270 sextants (accuracy: 62.9% 95%CI 56.3–69.4). PSMA PET ligands showed significantly higher sensitivity than mpMRI (p<0.001), whereas mpMRI demonstrated higher specificity (p=0.002). No significant differences were observed between [¹⁸F]F-PSMA-1007 and [⁶⁸Ga]Ga-PSMA-11. Detection of pathological features of local tumour extension was limited across all modalities. Sensitivity for EPE was12%, 23%, and 9%), and sensitivity for SVI was30%, 30%, and 14% for [¹⁸F]F-PSMA-1007 PET/CT, [⁶⁸Ga]Ga-PSMA-11 PET/CT, and mpMRI, respectively.

**Conclusion**

PSMA PET/CT ligands provide higher sensitivity than mpMRI for intraprostatic tumour localisation, while no statistically significant differences in diagnostic performance were detected between [¹⁸F]- and [⁶⁸Ga]-PSMA ligands in this cohort. These findings support the potential complementary role of PSMA PET and mpMRI for preoperative intraprostatic tumour localisationt in high-risk PC.

**Keywords:** [¹⁸F]F-PSMA-1007 PET/CT, [⁶⁸Ga]Ga-PSMA-11 PET/CT, Multiparametric MRI, Intraprostatic tumour localisation, Diagnostic accuracy study

The trial was registered in the EudraCT (European Union Drug Regulating Authorities Clinical Trials) database (EudraCT No. 2021-004846-39) in August 2021. URL: [Clinical Trials register - Search for 2021-004846-39](https://www.clinicaltrialsregister.eu/ctr-search/search?query=2021-004846-39)

**INTRODUCTION**

Prostate cancer (PC) is one of the most common malignancies among men and remains a major contributor to cancer-related morbidity and mortality[1]. Accurate assessment of intraprostatic tumour burden and local extension is essential for risk stratification and for guiding treatment, including selection of candidates for nerve-sparing surgery, focal therapy, or curative radiation[2]. Current clinical T-staging relies primarily on digital rectal examination, and although multiparametric MRI (mpMRI) is recommended for tumour localisation and surgical planning[2], its performance is limited by underestimation of tumour extent[3] and moderate sensitivity for detecting extraprostatic extension[4]. Although mpMRI is well established for prostate tumour detection and localisation, some clinically significant lesions may remain occult, and image quality and interpretation may be affected by artefacts arising from patient-related factors, including motion, hip prostheses, or prostatitis[5].

Prostate-specific membrane antigen (PSMA) PET is a highly sensitive molecular imaging modality widely used for staging in biochemical recurrence and newly diagnosed high-risk PC, particularly for nodal and distant metastases, but not recommended for primary intraprostatic T-staging[2, 6, 7]. Recent European Association of Urology-European Association of Nuclear Medicine (EAU-EANM) guidelines endorse its integration into treatment decision-making, supported by the EANM molecular imaging TNM (miTNM) classification[2, 8, 9]. The use of PSMA PET for miT‑staging appears to improve prognostic stratification compared with conventional work‑up, but the downstream clinical impact remains to be determined[2]. Both [⁶⁸Ga]- and [¹⁸F]-PSMA ligands demonstrate high tumour-to-background contrast and are increasingly investigated for intraprostatic tumour detection[10, 11]. [¹⁸F]-labelled compounds, such as [¹⁸F]F-PSMA-1007 PET/CT, offer theoretical advantages due to longer half-life, lower positron energy, and reduced urinary excretion[12].

Head-to-head comparisons of PSMA PET and mpMRI for intraprostatic tumour localisation and staging have shown inconsistent findings. Some studies report higher sensitivity with PSMA PET[13, 14], whereas others show no clear advantage over mpMRI; hybrid PET/mpMRI may provide complementary benefits[15-17]. Beyond conventional visual interpretation, recent advances in artificial intelligence and radiomics have enabled multimodal predictive models integrating PSMA PET and mpMRI. Studies combining [¹⁸F]F-PSMA-1007 PET with clinical, radiomic, and MRI features have demonstrated promising performance for predicting adverse pathological findings highlighting the complementary value of molecular and anatomical imaging biomarkers for individualized risk stratification[18]. However, these advanced approaches depend on reliable acquisition and interpretation of the individual imaging modalities, and direct comparison of different PSMA-ligands remains important before their incorporation into integrated predictive frameworks. Importantly, few studies have assessed multiple PSMA-ligands within the same cohort[19], and none has systematically performed region-based analyses comparing both [⁶⁸Ga]- and [¹⁸F]-labelled PSMA-ligands alongside mpMRI for intraprostatic tumour localisation. Consequently, the relative diagnostic contribution of individual PSMA-ligands and mpMRI requires further clarification using histopathological validation.

Therefore, we performed a prospective head-to-head comparison of [¹⁸F]F-PSMA-1007 PET/CT and [⁶⁸Ga]Ga-PSMA-11 PET/CT, with a secondary exploratory comparison including mpMRI, using whole-mount radical prostatectomy histopathology as the reference standard. The primary research question was whether [¹⁸F]F-PSMA-1007 PET/CT demonstrates similar diagnostic accuracy to [⁶⁸Ga]Ga-PSMA-11 PET/CT for detecting clinically significant prostate cancer at both the patient and region level. We hypothesised that the diagnostic accuracy of the two ligands would be similar. A secondary objective was to compare the diagnostic performance of both PSMA PET ligands with mpMRI for intraprostatic tumour localisation.

**MATERIALS AND METHODS**

**Study Design**

This prospective diagnostic accuracy study followed STARD guidelines [20]. It compared [⁶⁸Ga]Ga-PSMA-11 PET/CT and [¹⁸F]F-PSMA-1007 PET/CT for intraprostatic tumour detection using whole-mount histopathology of the entirely embedded prostatectomy specimen as the reference standard. The original study was designed as a comparative diagnostic accuracy study to compare the two PSMA PET ligands and was not powered or designed as an equivalence or non-inferiority trial. During the study, the protocol was amended to include mpMRI as an exploratory index test to allow comparison with the current standard imaging modality. Consequently, mpMRI was performed in a subset of patients, and all related analyses were considered exploratory.

**Approvals**

The study has been approved by the Danish Medical Counsel, the North Denmark Region Committee on Health Research Ethics (approval number: N-20200025), and all subjects signed an informed consent prior to inclusion. The study was registered in the EudraCT (European Union Drug Regulating Authorities Clinical Trials) database (registration number: 2021-004846-39). The study was conducted in accordance with Good Clinical Practice (GCP) guidelines and was monitored by the GCP Unit at Aalborg University Hospital, Denmark.

**Participants**

Consecutive men with high-risk, histologically confirmed prostate adenocarcinoma (EAU risk classification) were prospectively enrolled if they had no evidence of distant metastases on conventional imaging, including [⁶⁸Ga]Ga-PSMA-11 PET/CT, and were scheduled for robot-assisted radical prostatectomy after MDT review at Aalborg University Hospital (January 2022–July 2024). Exclusion criteria were prior PC treatment or another malignancy within the past five years (except non-melanoma skin cancer. This was a single-gate study in which all patients underwent both [⁶⁸Ga]Ga-PSMA-11 PET/CT and [¹⁸F]F-PSMA-1007 PET/CT before surgery, while mpMRI was performed in a subset of patients.

**Index tests**

**PSMA PET/CT Imaging**

All participants underwent clinically indicated [⁶⁸Ga]Ga-PSMA-11 PET/CT as part of standard care before study enrolment, followed by protocol-mandated [¹⁸F]F-PSMA-1007 PET/CT as a research scan.

**1.[⁶⁸Ga]Ga-PSMA-11** was locally synthesised at Aalborg University Hospital according to established protocols [21] and acquisition started 60 ± 10 minutes after injection of 2 MBq/kg on a Siemens Biograph mCT Flow (Siemens, Erlangen, Germany). Low-dose CT was used routinely, with contrast-enhanced CT performed in selected cases if no recent contrast CT was available.

**2. [¹⁸F]F-PSMA-1007** was produced under Good Manufacturing Practice conditions at Aarhus University Hospital and administered at 2 MBq/kg (150–300 MBq), with imaging performed 60 ± 10 minutes post-injection.

The interval between PET/CT scans was up to 1 month, and up to 3 months between imaging and prostatectomy was allowed.

**mpMRI (exploratory index test)**

mpMRI was added as an exploratory index test after protocol finalisation, and all related analyses were considered exploratory. Imaging was performed on a 3 Tesla scanner (Philips Ingenia Elition 3.0T X, Netherlands) using a pelvic phased-array coil. The protocol included T2-weighted imaging in three planes, diffusion-weighted imaging (DWI) with multiple b-values, apparent diffusion coefficient (ADC) mapping, and dynamic contrast-enhanced imaging (DCE-MRI).

**Reference Standard**

The reference standard was whole-mount histopathology of the prostatectomy specimen. Specimens were fixed in formalin for 48 hours. After removal of the seminal vesicles and proximal deferent ducts, the specimen was sectioned into 5-6 mm horizontal slices with additional 3–5 mm sagittal slices from the apical and basal slice. The specimen was entirely embedded in paraffin, using mega-blocks for horizontal slices, sectioned at 4–5 µm, and stained with H&E; immunohistochemistry was performed when necessary to confirm carcinoma. Histopathological examination served as the reference standard for classifying positive findings as malignant or benign. Tumour foci were mapped (Supplemental Figure S1), graded using Gleason patterns, and converted to ISUP grade according to the 2019 ISUP system[22]. Each specimen was independently evaluated by two blinded uropathologists, with consensus reached in case of disagreement. Findings were allocated to six prostate regions (left/right; base, mid, apex) and used as the reference standard. A region was classified as positive if it contained ISUP ≥3, or ISUP 2 with tertiary pattern 5, defining clinically significant PC (csPC). Given the multifocal nature of prostate cancer and our relatively small cohort, pathological assessment was based on the highest-grade tumor focus rather than any ISUP 2 lesion, providing the most clinically relevant reference standard for imaging-pathology correlation. A supplementary analysis using ISUP 2 lesions as an alternative reference criterion was also performed and is reported in the Supplementary Appendix. To minimise spatial mismatch between imaging and histopathology, the prostate was assessed separately in basal, mid-gland, and apical regions. The mid-gland was divided into multiple sections according to the individual prostate size with imaging assessed using the corresponding number of regions. Given uncertainties related to tissue deformation, specimen shrinkage, and the limited precision of matching imaging findings to 5 μm histological sections across a prostate spanning several centimetres, a neighbouring approach was applied. Tumour involvement in the most superior or inferior mid-gland section resulted in classification of the adjacent basal or apical region, respectively, as positive (Supplemental Figure S2).

**Blinding and Interpretation**

Each imaging modality ([⁶⁸Ga]Ga-PSMA-11 PET/CT, [¹⁸F]F-PSMA-1007 PET/CT, and mpMRI) was independently interpreted by two experienced readers with expertise in prostate imaging and PSMA PET interpretation. Equivocal findings were resolved by consensus and classified as positive or negative for cancer, and the consensus interpretation was used for final analysis. Individual reader assessments were not retained; therefore, inter-reader agreement was not evaluated. Readers were blinded to clinical data, histopathology, and other imaging modalities, and each reader interpreted only a single modality. Imaging findings were mapped to corresponding histopathological regions using a standardized worksheet for comparison. A sextant-based analysis was selected to facilitate direct comparison between imaging findings and whole-mount histopathology. Lesion localisation was determined based on anatomical landmarks, including the prostate base and apex, urethral position, and zonal anatomy. Lesions involving more than one anatomical region were assigned to each corresponding region. Given the multifocal nature of prostate cancer and the inherent challenges of precise lesion-to-lesion registration between in-vivo imaging and ex-vivo histopathology, sextant-based analysis with a predefined neighbouring-region approach provides a reproducible anatomical framework while minimizing registration bias.

**Test Positivity Criteria**

**PSMA PET/CT:** A positive finding was defined by visual assessment of PSMA uptake in the prostate based on focal tracer uptake exceeding the physiological background in prostate. In equivocal cases, a Prostate Cancer Molecular Imaging Standardized Evaluation score[9] (PROMISE- Criteria) ≥2 was considered positive. No SUVmax threshold, lesion size criteria, or predefined rules for index lesion selection were applied. For regional analysis, the prostate was divided into six anatomical regions, and each region was classified as positive if one or more suspicious lesions were identified. The same criteria were applied to both PSMA PET ligands, using the liver as the reference background for [⁶⁸Ga]Ga-PSMA-11 PET and the spleen as the reference background for [¹⁸F]PSMA-1007 PET as described in the PROMISE criteria[9]. Assessment of extraprostatic extension (EPE) and seminal vesicle invasion (SVI) was based on visual interpretation. EPE was considered present when focal tracer uptake extended beyond the prostatic contour with corresponding anatomical abnormalities on CT. SVI was defined as focal PSMA uptake involving the seminal vesicles with supportive anatomical correlation.

**mpMRI:** Lesions were scored according to the Prostate Imaging Reporting and Data System (PI-RADS), version 2.1[23]. Regions with a PI-RADS score ≥4 were classified as positive. EPE and SVI were assessed according to PI-RADS v2.1 criteria. EPE was considered present in cases with findings suggestive of extracapsular tumour extension, including capsular irregularity, bulging, neurovascular bundle involvement, loss of the rectoprostatic angle, or visible extension beyond the prostate. SVI was defined by tumour involvement of the seminal vesicles based on T2-weighted imaging, diffusion-weighted imaging, and contrast-enhanced sequences.

**Statistical analysis**

Continuous baseline characteristics were described by quartiles, and categorical by counts with percentages. Diagnostic accuracy metrics were calculated for the presence of csPC in prostate according to the histological examination as reference standard. To ensure comparability with earlier studies (16, 26), sensitivity, specificity and diagnostic accuracy was calculated based on 2 x 2 contingency tables at a patient level and on a region-level. For region-based analyses, we also used mixed-effects regression models with a random intercept for patient, treating sextant regions as repeated measurements within patients. Imaging modality was included as a fixed effect, and robust variance estimation was applied. Diagnostic accuracy estimates were reported with 95% confidence intervals, and differences between modalities were assessed using p values and 95% confidence intervals (α=0.05). The study was designed to detect differences in diagnostic performance between modalities and not to formally demonstrate equivalence or non-inferiority between PSMA ligands. True and false positive rates were derived from the regression model, with diagnostic measures calculated using the delta method. Missing data occurred only for mpMRI. Missing observations were handled using mixed-effects regression models, which provide valid estimates under the missing-at-random (MAR) assumption. Pairwise comparisons therefore included all available observations for the modalities being compared. Analyses were performed using Stata version 19.5 ((StataCorp LLC, College Station, TX, USA)).

**RESULTS**

**Study Population**

Between January 2022 and July 2024, 272 consecutive patients with biopsy-proven high-risk PC were screened at the multidisciplinary team confeerence at Aalborg University Hospital. Of these, 70 were eligible and 57 (81% of eligible; 21% of all screened) were enrolled (Figure 1). Fifty patients were included in the final analysis, all with complete histopathology available; 45 also underwent mpMRI. All patients underwent robot-assisted radical prostatectomy. [⁶⁸Ga]Ga-PSMA-11 PET/CT was performed first in all patients as part of routine clinical workflow, followed by [¹⁸F]F-PSMA-1007 PET/CT according to the study protocol. No adverse events related to [¹⁸F]F-PSMA-1007 administration or PET/CT imaging were observed. The median interval between the two PET/CT examinations was 9 days (IQR 9-21), and the intervals from PET/CT to radical prostatectomy were 21 days (IQR 16-28) for [⁶⁸Ga]Ga-PSMA-11 and 7 days (IQR 5-12) for [¹⁸F]F-PSMA-1007. Administered activities were 177 MBq (IQR 165-186) for [⁶⁸Ga]Ga-PSMA-11 and 220 MBq (IQR 194-240) for [¹⁸F]F-PSMA-1007, with median uptake times of 58 minutes (IQR 55-61) and 57 minutes (IQR 55-60), respectively. Baseline characteristics are shown in Table 1.

Patients assessed for eligibility (n=272)

Excluded (n = 215) 1. Not meeting inclusion criteria: n= 194 2. Declined participation: n = 13 3. Other reasons: n = 8

Patients included (n=57)

Withdrew before imaging (n=7) 1. Consent withdrawn: n=4 2. Technical/logistical: n=3

Final cohort (n=50) 1. [¹⁸F]F-PSMA-1007 PET/CT(research modality) : Completed (n=50) 2. [⁶⁸Ga]Ga-PSMA-11 PET/CT (clinical modality): Completed (n=50) 3. mpMRI (added after protocol): Exploratory subset (n=45)

Analysis Cohorts

PSMA paired per-protocol population: n = 50 Intention-to-diagnose population: n = 50

MRI analysis population (exploratory): n = 45

**Figure 1.** Flow of patient recruitment and analysis

**Detection of intraprostatic tumour: Per-patient analysis**

According to the histopathological reference standard, 66 tumours were identified in 49 patients with csPC, while one patient had no csPC according to our predefined histopathological threshold at final pathology. Using PROMISE criteria, [¹⁸F]F-PSMA-1007 PET/CT showed increased uptake in 49/50 patients (68 lesions; median (IQR) SUVmax 14.0 (10.0-21.0)), [⁶⁸Ga]Ga-PSMA-11 PET/CT in 50/50 patients (73 lesions; median (IQR) SUVmax 16.3 (9.9-24.5)), and mpMRI identified ≥PI-RADS 4 lesions in 43/45 patients (49 lesions). Patient-level sensitivity for csPC detection was 98% for [¹⁸F]F-PSMA-1007 PET/CT, 100% for [⁶⁸Ga]Ga-PSMA-11 PET/CT, and 96% for mpMRI. Overall diagnostic accuracy was 96%, 98%, and 93%, respectively. All PSMA-avid lesions without csPC corresponded to ISUP 1–2 disease on histopathology; thus, the apparent false-positive PET finding in the single patient without csPC reflected PSMA uptake in lower-grade prostate cancer rather than benign tissue. Using all ISUP grade group 2 lesions as the threshold for csPC, the diagnostic accuracy of [¹⁸F]F-PSMA-1007 PET/CT, [⁶⁸Ga]Ga-PSMA-11 PET/CT, and mpMRI was 98%, 100%, and 96%, respectively.

**Figure 2.** A 76-year-old man (patient 42) with biopsy-proven prostate cancer (Gleason score 4+3) and a PSA of 20 ng/mL at diagnosis. Transverse [¹⁸F]F-PSMA-1007 PET/CT (A) and [⁶⁸Ga]Ga-PSMA-11 PET/CT (B) demonstrate a lesion in the left anterior mid-gland and apex (red arrows), whereas T2-weighted mpMRI (C) shows no corresponding abnormality. Whole-mount histopathology confirmed a Gleason 4+3 adenocarcinoma in the same region.

**Detection of intraprostatic tumour: Per-region analysis**

csPC was present in 205 of 300 sextant regions. The neighbouring-region approach was applied in 16 regions. All regions were assessed with both PSMA PET/CT modalities, while 270 regions were evaluated with mpMRI. At the sextant level, [¹⁸F]F-PSMA-1007 PET/CT detected csPC in 143/300 regions, [⁶⁸Ga]Ga-PSMA-11 PET/CT in 136/300, and mpMRI in 89/270. Sensitivity/specificity were 69.8%/65.3% for ¹⁸F-PSMA, 66.3%/66.3% for ⁶⁸Ga-PSMA, and 48.6%/86.5% for mpMRI, with corresponding accuracies of 68.3%, 66.3%, and 61.1% (further details in Table 2). Accounting for the clustering of data, mixed-model estimates were calculated and showed similar results: sensitivity/specificity of 70.8%/67.6% ([¹⁸F]F-PSMA-1007 PET/CT), 67.4%/68.7% ([⁶⁸Ga]Ga-PSMA-11 PET/CT), and 50.9%/88.7% (mpMRI), with corresponding accuracies of 69.8%, 67.8%, and 62.9%, respectively (Table 3). No significant differences in overall accuracy were found between modalities (p=0.23). Pairwise comparisons did not identify statistically significant differences between the two PSMA PET ligands. Both PSMA-ligands were significantly more sensitive than mpMRI, while mpMRI had higher specificity (Table 4). Finally, the diagnostic accuracy in each of the sextant regions was calculated for each modality, treating each region as an independent variable, are provided in the Supplementary Material (supplemental table S1).

Similar to the per patient analysis, all analyses were repeated using a definition that included all ISUP grade group 2 lesions as positive. Detailed results are provided in the Supplementary Material (supplemental table S2 and S3).

**Detection of T3 tumours**

Extraprostatic extension (EPE) was present in 26 of 50 patients. Overall accuracy for EPE detection was 54.0% for [¹⁸F]F-PSMA-1007 PET/CT, 60.0% for [⁶⁸Ga]Ga-PSMA-11 PET/CT, and 53.3% for mpMRI (Table 5). Seminal vesicle invasion (SVI) was present in 10 patients (T3b). Sensitivity and specificity for SVI were 30.0%/100% for [¹⁸F]F-PSMA-1007 PET/CT, 30.0%/97.5% for [⁶⁸Ga]Ga-PSMA-11 PET/CT, and 14.3%/100% for mpMRI (Table 6).

**DISCUSSION**

In this prospective cohort of patients with high-risk PC, we compared [¹⁸F]F-PSMA-1007 PET/CT and [⁶⁸Ga]Ga-PSMA-11 PET/CT with mpMRI for detecting clinically significant intraprostatic tumours and T-staging using whole-mount histopathology as the reference standard. Both PSMA-ligands demonstrated high patient-level sensitivity and similardiagnostic accuracy estimates, with no statistically significant differences detected between modalities. At the regional level, both PSMA PET/CT tracers demonstrated higher sensitivity than mpMRI, while mpMRI showed higher specificity. Detection of EPE remained limited across all modalities, although [⁶⁸Ga]Ga-PSMA-11 PET/CT showed higher sensitivity than the other modalities. Detection of SVI showed low sensitivity across all modalities, despite high specificity, with no significant differences in diagnostic accuracy between modalities. Overall, PSMA PET/CT was more sensitive and mpMRI more specific. Both PSMA ligands provided similar diagnostic information in this cohort, while demonstrating complementary characteristics compared with mpMRI, in, supporting further evaluation of their role in preoperative assessment of high-risk PC.

Published comparative studies report substantial heterogeneity in the performance of PSMA-ligand PET and mpMRI for intraprostatic lesion detection[11]. Ren et al. reported [⁶⁸Ga]Ga-PSMA-11 PET/CT sensitivity 87% and specificity 80%, versus mpMRI 84% and 74%, based on pooled studies with mixed patient- and lesion-level analyses[24]. Wang et al. reported pooled sensitivities of 84% for both PSMA PET/CT and mpMRI, with specificities of 72% and 71%, without specification of index versus all lesions or lesion size thresholds[25]. Compared to our study, the variability is likely attributable to methodological differences, including selection criteria for regions or lesions, definitions of clinically significant disease, size thresholds, and approaches to lesion delineation and spatial matching, all of which are frequently underreported Although lesion-based analyses are commonly reported in PSMA PET studies, we adopted a sextant-based approach, as in previous studies[19], because the reference standard was whole-mount prostatectomy histopathology. Precise lesion-to-pathology matching is limited by tissue deformation after surgery, differences in spatial resolution, and the multifocal nature of prostate cancer. To facilitate comparison with previous studies, a supplementary sensitivity analysis including all ISUP grade group 2 lesions as positive for csPC was performed. Diagnostic estimates changed minimally, with PSMA PET retaining higher sensitivity and mpMRI higher specificity. Substantial heterogeneity between the results of this study and those of previous studies persisted, likely reflecting differences in study populations, reference standards, and methodological approaches.

Comparisons between PSMA ligands suggest subtle differences in diagnostic performance. In this study, [¹⁸F]F-PSMA-1007 and [⁶⁸Ga]Ga-PSMA-11 showed similar estimates of sensitivity, specificity, and overall accuracy for detecting intraprostatic csPCa; however, the study was not designed to establish equivalence or non-inferiority between the ligands. Although, prior limited literature reports modality-specific trends. For example, Kuten et al. found high accuracy for dominant lesion detection with both ligands in a head-to-head segment-based analysis, reporting higher sensitivity for [¹⁸F]F-PSMA-1007 (100%) but higher specificity for [⁶⁸Ga]Ga-PSMA-11 (98.2%)[19]. However, the study included a small cohort and did not focus specifically on clinically significant lesions or lesion size and did not use a neighbouring-region approach. A large meta-analysis was also unable to determine which ligand has superior diagnostic accuracy due to limited comparative data, although it suggested a trend favouring ¹⁸F-labelled ligands. Overall, single-ligand studies report a wide range of sensitivity and specificity[11]. Exterkate et al. reported segment-level tumor sensitivity of 60% and specificity of 93% for [¹⁸F]F-PSMA-1007 [26]. Mookerji et al. reported 87% sensitivity for all lesions [¹⁸F]F-PSMA-1007 relative to pathology[27]. For [⁶⁸Ga]Ga-PSMA-11, Mai et al. reported 92.9% sensitivity for index lesions[28]. As these studies did not perform direct head-to-head comparisons of [¹⁸F]- and [⁶⁸Ga]-PSMA ligands, and because of methodological heterogeneity—including differences in region- versus lesion-based analyses, application of size thresholds, and lack of standardized methods for delineating neighboring regions—there remains insufficient evidence to draw a definitive conclusion regarding the superior ligand. The present study support that [¹⁸F]F-PSMA-1007 and [⁶⁸Ga]Ga-PSMA-11 have similar diagnostic performance for the detection of csPC within this cohort.

All imaging modalities in our study demonstrated modest accuracy for detecting EPE and good accuracy for detecting SVI (>0.80). In a previous meta-analysis, we reported an accuracy of 73% for PSMA PET/CT in detecting EPE and 87% for SVI[11]. Wang et al. reported PSMA PET sensitivity and specificity for EPE of 61% and 74%, and for mpMRI 67% and 77%; for SVI, PSMA PET sensitivity and specificity were 62% and 90%, and mpMRI 60% and 92%[25]. Compared with these studies, our cohort demonstrated lower sensitivity but comparable specificity for both EPE and SVI across all three imaging modalities. Such differences are to be expected and are commonly reported in single-centre or smaller studies, in contrast to meta-analyses that aggregate data from multiple centres and tracers.

The present study has several strengths including its prospective design, a well-characterised cohort from a high-volume centre, and use of whole-mount histopathology as the reference standard. It also enables direct comparison of [¹⁸F]F-PSMA-1007 and [⁶⁸Ga]Ga-PSMA-11 PET/CT within the same population, and applies a mixed-effects model to account for intra-patient clustering, improving robustness of the diagnostic estimates. Limitations include its single-centre design, which may limit generalisability, and the absence of a formal sample size calculation that may be underpowered for small subgroup differences. Furthermore, the study was not designed as an equivalence or non-inferiority trial; therefore, the absence of statistically significant differences between the two PSMA-ligands should not be interpreted as evidence of equivalence or interchangeability. The relatively small sample size may have limited the ability to detect modest differences in diagnostic performance. Reader variability and lack of fully standardised interpretation criteria may also have influenced results. The lower sensitivity for EPE and SVI compared with meta-analyses may relate to smaller lesions and absence of standardised criteria such as capsular contact length for EPE assessment[16]. Selection bias may be present, as patients from a high-volume tertiary centre may not reflect the general population and could have more complex or higher-risk disease. Finally, although recent multimodal radiomics and deep-learning models integrating PSMA PET with mpMRI have shown promising results for predicting adverse pathology after radical prostatectomy[18], our study evaluated the imaging modalities individually rather than as an integrated diagnostic strategy. In addition, outcome-studies are needed to assess how PSMA PET–guided management influences surgical planning, focal therapy, and long-term oncological outcomes, to clarify its role in routine T-staging. Furthermore, the PSMA PET ligands comparison was prespecified, whereas analyses involving mpMRI were secondary and exploratory following a protocol amendment.

In conclusion PSMA PET/CT performed favourably compared with mpMRI for intraprostatic tumour localisation in high-risk PC with both [¹⁸F]- and [⁶⁸Ga]-labelled ligands demonstrating no statistically significant differences in diagnostic accuracy; however, these findings do not establish equivalence or interchangeability between the two PSMA-ligands. These findings provide hypothesis-generating evidence that PSMA PET and mpMRI may offer complementary diagnostic information in the preoperative assessment of high-risk prostate cancer. As emerging multimodal imaging models increasingly integrate PSMA PET with mpMRI for individualized risk stratification, accurate characterization of the diagnostic accuracy of individual imaging modalities remains an essential prerequisite for their future clinical implementation.

**REFERENCES**

1.	Siegel RL, Kratzer TB, Giaquinto AN, Sung H, Jemal A. Cancer statistics, 2025. CA Cancer J Clin. 2025;75:10–45. doi:10.3322/caac.21871.

2.	Cornford P, van den Bergh RCN, Briers E, Van den Broeck T, Brunckhorst O, Darraugh J, et al. EAU-EANM-ESTRO-ESUR-ISUP-SIOG Guidelines on Prostate Cancer-2024 Update. Part I: Screening, Diagnosis, and Local Treatment with Curative Intent. Eur Urol. 2024;86:148–63. doi:10.1016/j.eururo.2024.03.027.

3.	Pooli A, Johnson DC, Shirk J, Markovic D, Sadun TY, Sisk AE, Jr., et al. Predicting Pathological Tumor Size in Prostate Cancer Based on Multiparametric Prostate Magnetic Resonance Imaging and Preoperative Findings. The Journal of urology. 2021;205:444–51. doi:10.1097/JU.0000000000001389.

4.	de Rooij M, Hamoen EH, Witjes JA, Barentsz JO, Rovers MM. Accuracy of Magnetic Resonance Imaging for Local Staging of Prostate Cancer: A Diagnostic Meta-analysis. Eur Urol. 2016;70:233–45. doi:10.1016/j.eururo.2015.07.029.

5.	Lin Y, Yilmaz EC, Belue MJ, Turkbey B. Prostate MRI and image Quality: It is time to take stock. Eur J Radiol. 2023;161:110757. doi:10.1016/j.ejrad.2023.110757.

6.	Lowrance W, Dreicer R, Jarrard DF, Scarpato KR, Kim SK, Kirkby E, et al. Updates to Advanced Prostate Cancer: AUA/SUO Guideline (2023). The Journal of urology. 2023;209:1082–90. doi:10.1097/JU.0000000000003452.

7.	Schaeffer EM, Srinivas S, Adra N, An Y, Bitting R, Chapin B, et al. NCCN Guidelines(R) Insights: Prostate Cancer, Version 3.2024. J Natl Compr Canc Netw. 2024;22:140–50. doi:10.6004/jnccn.2024.0019.

8.	Ceci F, Oprea-Lager DE, Emmett L, Adam JA, Bomanji J, Czernin J, et al. E-PSMA: the EANM standardized reporting guidelines v1.0 for PSMA-PET. European journal of nuclear medicine and molecular imaging. 2021;48:1626–38. doi:10.1007/s00259-021-05245-y.

9.	Seifert R, Emmett L, Rowe SP, Herrmann K, Hadaschik B, Calais J, et al. Second Version of the Prostate Cancer Molecular Imaging Standardized Evaluation Framework Including Response Evaluation for Clinical Trials (PROMISE V2). Eur Urol. 2023;83:405–12. doi:10.1016/j.eururo.2023.02.002.

10.	Ma J, Yang Q, Ye X, Xu W, Chang Y, Chen R, et al. Head-to-head comparison of prostate-specific membrane antigen PET and multiparametric MRI in the diagnosis of pretreatment patients with prostate cancer: a meta-analysis. European radiology. 2024;34:4017–37. doi:10.1007/s00330-023-10436-2.

11.	Gossili F, Mogensen AW, Konnerup TC, Bouchelouche K, Alberts I, Afshar-Oromieh A, et al. The diagnostic accuracy of radiolabeled PSMA-ligand PET for tumour staging in newly diagnosed prostate cancer patients compared to histopathology: a systematic review and meta-analysis. European journal of nuclear medicine and molecular imaging. 2023;51:281–94. doi:10.1007/s00259-023-06392-0.

12.	Foley RW, Redman SL, Graham RN, Loughborough WW, Little D. Fluorine-18 labelled prostate-specific membrane antigen (PSMA)-1007 positron-emission tomography-computed tomography: normal patterns, pearls, and pitfalls. Clin Radiol. 2020;75:903–13. doi:10.1016/j.crad.2020.06.031.

13.	Kivikallio A, Malaspina S, Saarinen I, Seppanen M, Anttinen M, Jambor I, et al. Prospective comparison of (18)F-PSMA-1007 PET/CT and MRI with histopathology as the reference standard for intraprostatic tumour detection and T-staging of high-risk prostate cancer. European journal of nuclear medicine and molecular imaging. 2025;52:3709–19. doi:10.1007/s00259-025-07208-z.

14.	Talavera Cobo V, Yanez Ruiz CA, Tapia Tapia MD, Calva Lopez A, Munoz Bastidas CA, Ancizu Marckert FJ, et al. Diagnostic Accuracy of PSMA-PET/CT vs. mpMRI in Primary Staging of Intermediate- and High-Risk Prostate Cancer. Med Sci (Basel). 2026;14. doi:10.3390/medsci14010064.

15.	Sonni I, Felker ER, Lenis AT, Sisk AE, Bahri S, Allen-Auerbach M, et al. Head-to-Head Comparison of (68)Ga-PSMA-11 PET/CT and mpMRI with a Histopathology Gold Standard in the Detection, Intraprostatic Localization, and Determination of Local Extension of Primary Prostate Cancer: Results from a Prospective Single-Center Imaging Trial. Journal of nuclear medicine : official publication, Society of Nuclear Medicine. 2022;63:847–54. doi:10.2967/jnumed.121.262398.

16.	Hvittfeldt E, Hedeer F, Thimansson E, Sandeman K, Minarik D, Ingvar J, et al. Semi-standardized evaluation of extraprostatic extension and seminal vesicle invasion with [(18)F]PSMA-1007 PET/CT: a comparison to MRI using histopathology as reference. EJNMMI Rep. 2025;9:1. doi:10.1186/s41824-024-00234-4.

17.	Muehlematter UJ, Burger IA, Becker AS, Schawkat K, Hötker AM, Reiner CS, et al. Diagnostic Accuracy of Multiparametric MRI versus (68)Ga-PSMA-11 PET/MRI for Extracapsular Extension and Seminal Vesicle Invasion in Patients with Prostate Cancer. Radiology. 2019;293:350–8. doi:10.1148/radiol.2019190687.

18.	Lin H, Yao F, Yi X, Yuan Y, Xu J, Chen L, et al. Prediction of adverse pathology in prostate cancer using a multimodal deep learning approach based on [(18)F]PSMA-1007 PET/CT and multiparametric MRI. European journal of nuclear medicine and molecular imaging. 2025;52:2814–25. doi:10.1007/s00259-025-07134-0.

19.	Kuten J, Fahoum I, Savin Z, Shamni O, Gitstein G, Hershkovitz D, et al. Head-to-Head Comparison of Ga-68-PSMA-11 with F-18-PSMA-1007 PET/CT in Staging Prostate Cancer Using Histopathology and Immunohistochemical Analysis as a Reference Standard. Journal of Nuclear Medicine. 2020;61:527–32. doi:10.2967/JNUMED.119.234187.

20.	Cohen JF, Korevaar DA, Altman DG, Bruns DE, Gatsonis CA, Hooft L, et al. STARD 2015 guidelines for reporting diagnostic accuracy studies: explanation and elaboration. BMJ Open. 2016;6:e012799. doi:10.1136/bmjopen-2016-012799.

21.	Nielsen JB, Zacho HD, Haberkorn U, Nielsen KM, Dettmann K, Langkilde NC, et al. A Comprehensive Safety Evaluation of 68Ga-Labeled Ligand Prostate-Specific Membrane Antigen 11 PET/CT in Prostate Cancer: The Results of 2 Prospective, Multicenter Trials. Clin Nucl Med. 2017;42:520–4. doi:10.1097/RLU.0000000000001681.

22.	van Leenders G, van der Kwast TH, Grignon DJ, Evans AJ, Kristiansen G, Kweldam CF, et al. The 2019 International Society of Urological Pathology (ISUP) Consensus Conference on Grading of Prostatic Carcinoma. Am J Surg Pathol. 2020;44:e87–e99. doi:10.1097/PAS.0000000000001497.

23.	Turkbey B, Rosenkrantz AB, Haider MA, Padhani AR, Villeirs G, Macura KJ, et al. Prostate Imaging Reporting and Data System Version 2.1: 2019 Update of Prostate Imaging Reporting and Data System Version 2. Eur Urol. 2019;76:340–51. doi:10.1016/j.eururo.2019.02.033.

24.	Ren X, Nur Salihin Yusoff M, Hartini Mohd Taib N, Zhang L, Wang K. (68)Ga-prostate specific membrane antigen-11 PET/CT versus multiparametric MRI in the detection of primary prostate cancer: A systematic review and head-to-head comparative meta-analysis. Eur J Radiol. 2024;170:111274. doi:10.1016/j.ejrad.2023.111274.

25.	Wang YF, Lo CY, Chen LY, Chang CW, Huang YT, Huang YY, et al. Comparing the Detection Performance Between Multiparametric Magnetic Resonance Imaging and Prostate-Specific Membrane Antigen PET/CT in Patients With Localized Prostate Cancer: A Systematic Review and Meta-analysis. Clin Nucl Med. 2023;48:e321–e31. doi:10.1097/RLU.0000000000004646.

26.	Exterkate L, Hermsen R, Kusters-Vandevelde HVN, Prette JF, Baas DJH, Somford DM, et al. Head-to-Head Comparison of (18)F-PSMA-1007 Positron Emission Tomography/Computed Tomography and Multiparametric Magnetic Resonance Imaging with Whole-mount Histopathology as Reference in Localisation and Staging of Primary Prostate Cancer. Eur Urol Oncol. 2023;6:574–81. doi:10.1016/j.euo.2023.04.006.

27.	Mookerji N, Pfanner T, Hui A, Huang G, Albers P, Mittal R, et al. Fluorine-18 Prostate-Specific Membrane Antigen-1007 PET/CT vs Multiparametric MRI for Locoregional Staging of Prostate Cancer. JAMA Oncol. 2024;10:1097–103. doi:10.1001/jamaoncol.2024.3196.

28.	Mai Z, Zhu M, Feng T, Zhou Z, Zhou Y, Wang D, et al. Comparisons of mpMRI, (68)Ga-PSMA PET/CT and mpMRI combined with (68)Ga-PSMA PET/CT in diagnosing prostate cancer based on tumor detection, localization and staging. World journal of urology. 2024;42:29. doi:10.1007/s00345-023-04729-w.

**Statements & Declarations**

**Funding**

This work was supported by Research funding of Nuclear Medicine Deparment, Aalborg University Hospital and author F.G. has received research support from Aalborg University Hospital.

**Competing Interests**

The authors have no relevant financial or non-financial interests to disclose.

**Author Contributions**

The study was conceptualised by Helle D. Zacho and Farid Gossili. The methodology was developed by Farid Gossili with support from Kirsten Bouchelouche and Helle D. Zacho. Farid Gossili prepared the study materials with support from Helle D. Zacho. Patient recruitment was undertaken by Anja Morratz Laursen, Farid Gossili, and Helle D. Zacho. Data collection was performed by Farid Gossili, Frederik Harving, Astrid Christine Petersen, Claus Madsen, Kirsten Bouchelouche, Robert Johannes Leusink, Ayesha Ahmed, and Helle D. Zacho. Data analysis was conducted by Niels Henrik Bruun and Farid Gossili. The initial draft of the manuscript was written by Farid Gossili. All authors critically revised the manuscript, contributed to intellectual content, and approved the final version.

**Data Availability**

The datasets generated during and/or analysed during the current study are available from the corresponding author on reasonable request.

**Ethics approval**

This study was performed in line with the principles of the Declaration of Helsinki. Approval was granted by the Danish Medical Counsel, the North Denmark Region Committee on Health Research Ethics (approval number: N-20200025).

**Consent to participateand publish**

Informed consent was obtained from all individual participants included in the study.

**Supplementary Information**

The online version contains supplementary material.
